# Effects of core training on balance performance in older adults: a systematic review and meta-analysis

**DOI:** 10.3389/fpubh.2025.1661460

**Published:** 2025-10-09

**Authors:** Yuanji Zhong, Wenhao Guo, Pengwei Chen, Yongshun Wang

**Affiliations:** ^1^School of Physical Education and Arts, Jiangxi University of Science and Technology, Ganzhou, Jiangxi, China; ^2^School of Recreational Sports and Tourism, Beijing Sport University, Beijing, China; ^3^College of Physical Education, Huaqiao University, Quanzhou, China

**Keywords:** core training, balance, older adults, fall prevention, meta-analysis

## Abstract

**Background:**

Core training (CT) has been increasingly recognized as a promising intervention for improving balance in older adults, a demographic particularly susceptible to falls and fall-related injuries. This systematic review and meta-analysis sought to evaluate the efficacy of CT on balance in older adults, providing substantial evidence to establish its role in fall prevention strategies.

**Methods:**

A comprehensive and systematic search of multiple databases, including PubMed, Cochrane Library, Web of Science, EBSCOhost, Embase and Google Scholar, was conducted to identify relevant studies. Eligible studies included single-group trials or randomized controlled trials that assessed the impact of CT on balance outcomes in healthy older adults (aged ≥60 years). It utilized the Cochrane Risk of Bias 2 (RoB 2) tool to assess the risk of bias across all included studies. Data were extracted from eleven studies and analyzed using Review Manager software version 5.4 and Stata 17.0, evaluating both dynamic and static balance parameters.

**Results:**

The findings revealed statistically significant improvements in both dynamic and static balance metrics, including Gait Test (GT) (SMD = 0.32; 95% CI = 0.02, 0.63; *p* < 0.05), Functional Reach Test (FRT) (SMD = 0.82; 95% CI = 0.50, 1.24; *p* < 0.00001), Timed Up and Go (TUG) test (SMD = −0.81; 95% CI = −1.62, 0.00; *p* = 0.05), and One-Leg Stance Test (OLST) (MD = 3.19; 95% CI = 1.74, 4.64; *p* < 0.001). Subgroup analyses further indicated that CT had the most significant effect on dynamic balance, particularly in FRT. Additionally, longer intervention durations (≥45 min) resulted in more pronounced benefits for dynamic balance compared to shorter sessions. CT demonstrated superior effects on GT compared to Pilates Training (PT).

**Conclusion:**

CT is a highly effective intervention for enhancing balance in older adults, supporting its integration into fall prevention programs. However, given the heterogeneity across studies, further rigorously designed trials with standardized intervention protocols and outcome measures are necessary to confirm the long-term benefits and optimal parameters for balance enhancement.

**Systematic review registration:**

Inplasy.com, INPLASY202412006.

## 1 Introduction

Population aging has emerged as an unprecedented global challenge, with profound implications for health systems and societal structures. Projections from the World Health Organization (WHO) indicate that by 2030, individuals aged 60 years and older will constitute one-sixth of the global population (1). This dramatic demographic shift has brought “healthy aging” to the forefront of global policy agendas, emphasizing the need to promote well-being and functional independence among older adults. As individuals age, physiological changes, such as reductions in muscle strength and bone density, undermine the integrity of the musculoskeletal system and compromise joint stability. These alterations significantly elevate the risk of falls, which are not only a leading cause of injury but also the second most common cause of unintentional injury-related mortality worldwide (2, 3). Among older adults, falls pose an especially serious threat, accounting for a substantial proportion of injuries and fatalities. Epidemiological data reveal that the incidence of falls ranges from 28 to 35% among those aged 60 and older, with the prevalence increasing to 42% in individuals over 70 years of age (4). Given the considerable health and economic burdens associated with falls in the aging population, the urgency of developing effective fall prevention strategies cannot be overstated. Identifying and implementing interventions that address fall risk factors is paramount to safeguarding the health, autonomy, and quality of life of older adults while alleviating the strain on healthcare systems.

Balance is a critical factor in fall prevention, playing a fundamental role in maintaining stability and mobility. It is defined as the ability to maintain the body’s center of mass within its base of support, achieved through the seamless integration of the visual, vestibular, and proprioceptive systems (5). This coordination ensures stability under both static and dynamic conditions, making balance a critical determinant of physical functionality in older adults. Among the myriad factors influencing falls in the older population, the enhancement of balance is widely regarded as a cornerstone strategy for improving postural stability and mitigating fall risks (6). Declines in balance, often associated with aging, have prompted an increasing volume of research dedicated to identifying effective interventions aimed at bolstering balance performance in older adults (7). Among these approaches, CT has emerged as a highly targeted and efficacious solution, garnering significant attention for its role in enhancing postural control and reducing fall susceptibility (8, 9).

CT primarily focuses on enhancing the stability, coordination, and functional capacity of the trunk muscles, including the abdominal muscles, erector spinae, and iliopsoas (10). For instance, Swiss ball core strengthening exercises have been demonstrated to yield significant improvements in spinal stability and overall muscular strength (11). Evidence suggests that, compared to younger populations, older adults experience more pronounced improvements in balance and gait performance following CT, a phenomenon likely attributable to the decline in balance associated with aging (2). Despite a growing body of literature demonstrating that CT can effectively enhance balance, reduce fall risk, and decrease fall incidence in older adults (8, 9, 12, 13), several studies are hindered by limitations such as small sample sizes, inconsistent intervention protocols, and variability in outcome measures, which undermine the generalizability of their findings. Systematic review and meta-analysis, an advanced methodological approach for systematically synthesizing and quantifying the results of multiple studies, offers a robust solution to these limitations (14). Specifically, systematic review and meta-analysis facilitates the aggregation of data across studies, calculation of effect sizes, and generation of robust evidence for clinical practice and policy development (15). Therefore, conducting a meta-analysis to evaluate the effects of CT on balance in older adults is essential to provide comprehensive and actionable insights for clinical and public health applications.

In recent years, numerous systematic reviews and meta-analyses have explored the effects of CT and related physical activities on balance outcomes in older adults. These studies have investigated the effects of interventions such as PT (16–20), Tai Chi (21, 22), balance training (23), and resistance training (24), which share certain similarities with CT in targeting balance enhancement. However, unlike these interventions, CT is unique in its training modalities and objectives, potentially leading to different effects on balance in older adults. More importantly, CT often serves as the foundational component of these exercise regimes (25), yet research specifically examining its impact on balance in older adults is limited. Although a systematic review and meta-analysis examining the effects of CT on balance in older adults has been conducted (26), the findings were limited to samples from Chinese populations. These results, which emphasize the effectiveness of CT in improving dynamic balance and reducing fall risk, may not be generalizable to other populations. Cultural, lifestyle, and physiological differences between regions may significantly influence exercise outcomes, highlighting the need for studies incorporating diverse populations. Consequently, while existing research highlights the benefits of CT in improving balance among older adults, further studies are required to assess its applicability across diverse demographic and cultural settings. This would provide more comprehensive insights into its global relevance and clinical utility.

This meta-analysis evaluates the impact of CT on balance performance in older adults and examines factors contributing to heterogeneity across studies. Additionally, it reviews the existing literature, highlights methodological and contextual limitations, and provides actionable recommendations for future studies. The findings provide critical insights for older adult health management, the design of exercise interventions, and the development of evidence-based public health policies. These findings will inform clinical practices and assist policymakers in formulating targeted strategies to prevent falls and enhance the quality of life among older adults.

## 2 Methods

There was adherence to the Preferred Reporting Items for Systematic Reviews and Meta-Analyses (PRISMA) guidelines throughout the entire process of conducting this systematic review (27), and the review protocol has been registered on Inplasy.com (INPLASY202412006).

### 2.1 Search strategy

Prominent academic databases were considered to search the related literature, including PubMed, EBSCOhost, Embase, Cochrane Library, Web of Science, as well as supplementary searches through the Google Scholar search engine, until 30th Nov 2024. For each independent database, a strategic search query was conducted by the title and abstract (Supplementary Table S1). The primary keywords considered for gathering related studies were: (“core strength training” OR “core-muscle training” OR “core training” OR “core-stability exercise” OR “core exercise”) AND (“balance” OR “static balance” OR “dynamic balance” OR “functional balance” OR “postural balance”) AND (“old” OR “aged” OR “senior” OR “elder”).

### 2.2 Eligibility criteria

The literature search was conducted using the PICOS framework. To be deemed eligible, studies were required to satisfy all the inclusion criteria specified (Table 1). The inclusion criteria were: (a) participants were healthy older adults with a mean age ≥ 60 years; (b) CT should be specifically isolated and explicitly discussed, with a minimum intervention duration of 4 weeks; (c) the comparison in studies should be either single-group or multiple-group trials; (d) The study outcomes must include the assessment of at least one behavioral balance outcome; (e) articles must be written in English and adhere to an experimental design, including single-group trials or randomized controlled trials.

**Table 1:** PICOS eligibility criteria.

PICOS	Detailed information
Population	Healthy older adults
Intervention	Core training (not less than 4 weeks)
Comparison	Two or more groups and single-group trials
Outcome	At least one behavioral balance outcome (e.g., gait speed)
Study design	Single-group Trials or Randomized Controlled Trials

Studies were excluded if they met any of the following conditions: (a) non-original or grey literature, including reviews, dissertations, conference abstracts, or technical reports, as these sources generally lack peer review and standardized reporting, which may increase the risk of bias; (b) absence of outcome indicators directly related to balance performance; (c) duplicate publications or repeated analyses, in which case the most recent or methodologically rigorous version was retained; (d) unavailability of the full text, which prevented quality assessment and data extraction; (e) articles not published in English, excluded to maintain methodological consistency and avoid misinterpretation due to language barriers; (f) insufficient data reporting, specifically studies that did not provide both mean and standard deviation for balance outcomes and where such information could not be extracted or obtained from the authors; (g) study populations involving older adults with conditions such as significant cognitive impairments (e.g., Alzheimer’s disease, dementia), major orthopedic disorders (e.g., lower limb fractures), neurological diseases (e.g., stroke, Parkinson’s disease), or other comorbidities that substantially restricted mobility; and (h) unpublished studies, which often lack methodological transparency and accessible data, making them unsuitable for reliable meta-analytic synthesis.

### 2.3 Selection process

**Figure 1:**
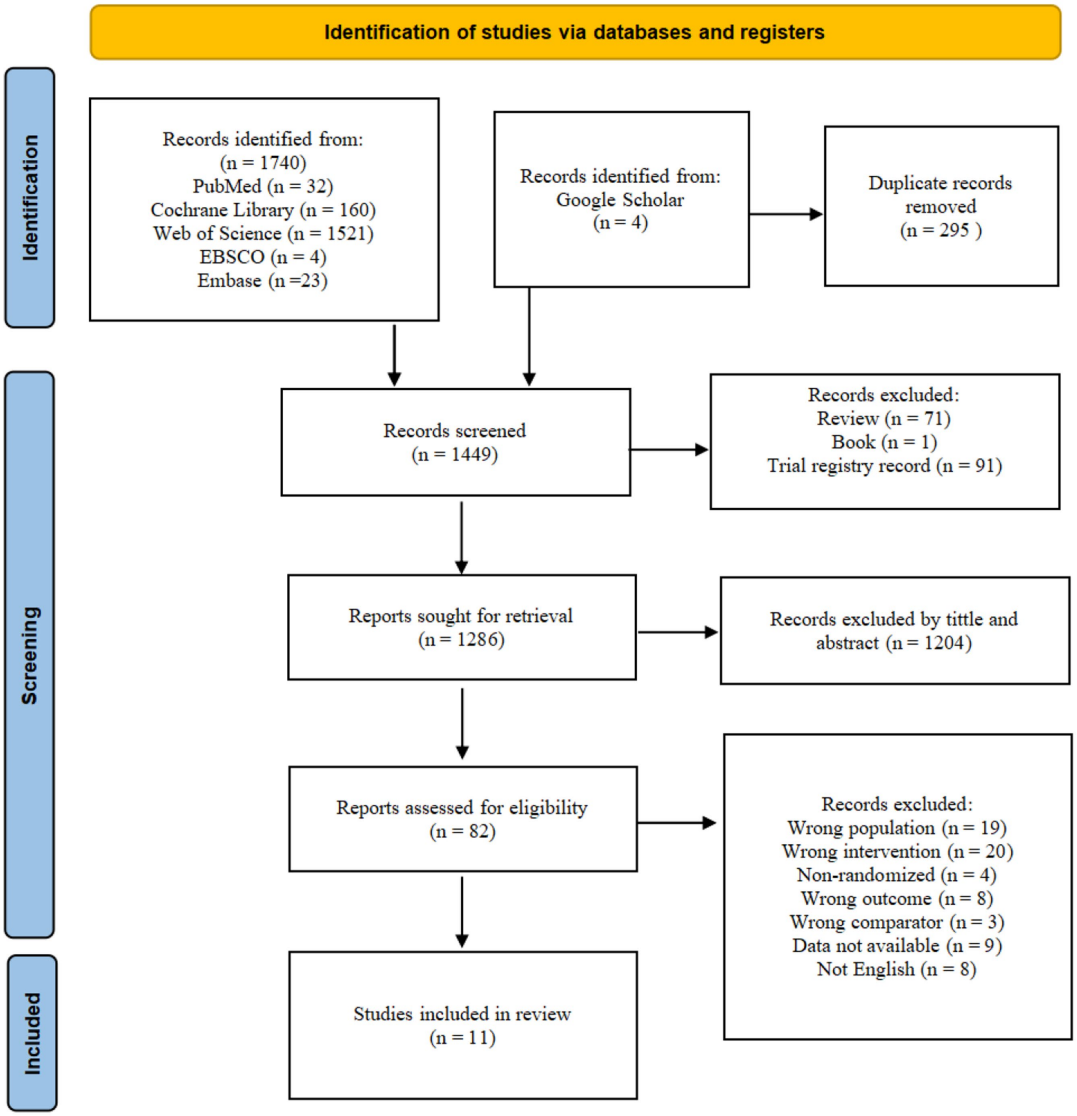
Summary Preferred Reporting Items for Systematic Reviews and Meta-Analyses (PRISMA) flowchart identifying the study selection process.

All records retrieved from the databases were imported into reference management software (EndNote X9). An independent researcher (YZ) identified and removed duplicate entries. The remaining records were subsequently screened according to predefined inclusion and exclusion criteria. A two-step screening process was conducted by the primary researchers (WG and YZ). The first step involved an initial assessment based on titles and abstracts, followed by a second phase of full-text screening. Study eligibility was evaluated using the PICOS framework. Figure 1 illustrates the details of the selection procedure.

### 2.4 Data extraction

Data extraction was conducted independently by two authors (WG and PC) using a standardized data extraction form. The extracted information included the general characteristics of the studies (authors, publication year, and country where the study was conducted), participant characteristics (age, gender, and the number of participants in each group), detailed information about the intervention group (CT type, frequency, intensity, and duration), detailed information about the control group (type, frequency, intensity, and duration of training), outcome measures, and primary results. Any discrepancies during the extraction process were resolved through discussion between the authors.

### 2.5 Risk of bias

Two reviewers (YZ and WG) independently appraised the risk of bias for each included study using the Cochrane Risk of Bias 2 (RoB 2) tool (28). The assessment covered five domains: the randomization process, deviations from intended interventions, missing outcome data, measurement of outcomes, and selection of the reported result. Prior to the formal assessment, the reviewers completed a calibration exercise on a subset of studies to harmonize interpretations of RoB 2 signaling questions and to agree on explicit decision rules for domain-level judgments. Discrepancies at the item or domain level were reconciled through structured discussion with reference to the RoB 2 guidance. If consensus could not be reached, a third author (PC) was consulted to conduct further evaluation and provide the final judgment. All decisions were documented with concise rationales, and overall risk-of-bias ratings were derived from domain-level judgments according to the RoB 2 algorithm.

### 2.6 Data analysis

Balance outcomes were categorized into static balance, dynamic balance, or a combination of both. Dynamic balance test (DBT) was defined as the ability to maintain stability while moving through space, regardless of whether foot movement was involved (e.g., GT, FRT, TUG Test). Static balance test (SBT) referred to the ability to maintain an upright posture with both feet in full contact with the ground (e.g., OLST, Balance Board Test). Studies included in the meta-analysis were required to report outcome measures that effectively reflected changes in balance.

The mean (M), standard deviation (SD), and sample size (N) of the outcome measures from the included studies were recorded. These data, based on either post-intervention values for the experimental and control groups or pre- and post-intervention differences, were recorded in Microsoft Excel (Microsoft 2016, Santa Rosa, California) for meta-analysis. Statistical analyses of the outcome measures were conducted using Review Manager software version 5.4.

Initially, a heterogeneity test was performed using the I^2^ statistic to evaluate the degree of heterogeneity across studies. According to the Cochrane Handbook, no heterogeneity is indicated when I^2^ = 0, and a fixed-effects model is employed for I^2^ < 50%. Conversely, a random-effects model is used if I^2^ > 50%. For studies with consistent outcome measurement units, mean difference (MD) was used as the effect size; otherwise, standardized mean difference (SMD) was applied. Both were calculated with 95% confidence intervals, and results were considered statistically significant at *p* < 0.05. Subgroup analyses were conducted to explore the sources of heterogeneity in the effects of CT on balance outcomes. Finally, the present study employed Begg’s rank correlation method and Egger’s linear regression method within Stata 17.0 to assess publication bias, with trim and fill methods applied to address any significant publication bias detected (29, 30).

## 3 Results

### 3.1 Study selection

Figure 1 presents the PRISMA flow diagram, illustrating the detailed screening process. An initial database search identified a total of 1,740 articles, with 4 additional articles retrieved from Google Scholar. After removing duplicates, reviews, books, and trial records, 1,286 articles remained for screening based on titles and abstracts. Subsequently, 82 full-text articles were reviewed for eligibility, resulting in the inclusion of 11 studies (12, 31–40).

### 3.2 Study characteristics

The studies reviewed, published between 2005 and 2024, span a period of 20 years, with their characteristics summarized in Table 2. These studies were conducted in various geographical regions, including Italy, Germany, South Korea, the United States, Spain, France, and Iran, collectively involving 442 older adults. The key characteristics of the studies are as follows: (1) Gender: Four studies specifically focused on female participants (32, 34, 37, 38), while the remaining studies included mixed-gender samples. (2) Age: All studies reported the age of participants, with an age range of 60 to 85 years identified across the eight studies. (3) Interventions: In terms of interventions, eight studies employed CT methods, such as core muscle training, core instability strength training, core resistance training, or core stability training (12, 31–35, 39, 40). The remaining three studies incorporated Pilates-based CT approaches (36–38). (4) Training frequency: All studies reported the frequency of training sessions, which ranged from 1 to 3 sessions per week. (5) Duration per Session: The duration of each training session varied between 20 and 60 min across all studies. (6) Intervention duration: All included studies reported the total intervention duration, ranging from 6 to 18 weeks. (7) Outcome measures: The studies predominantly employed well-established balance assessment methods, such as GT (32, 33, 36, 37), the TUG test (33, 35, 38), FRT (12, 31, 33, 36), and OLST (32, 35, 38).

**Table 2:** Study characteristics.

Study	Population	Gender	Age	Intervention group	Control group	Duration	Frequency of the intervention	Measurement tools	Outcome
Kahle 2014 (31), USA	IG: 12 CG: 12	M/F	IG/CG: 65–85	CT	Routine training	6 weeks	Three times a week; 20-35 min	FRT, SEBT	FRT↑, SEBT↑
Granacher 2013 (33), Germany	IG: 16 CG: 16	M/F	IG: 70.8 ± 4.1 CG: 70.2 ± 4.5	CT	Routine training	9 weeks	Twice a week; 60 min	DBT (SV, FRT), FM (TUG)	DBT (GT↑, FRT↑), FM (TUG ↓)
Markovic 2015 (34), France	IG: 17 CG: 17	F	IG/CG: 70 ± 4	Huber device with CT	PT	8 weeks	Three times a week; 60 min	SBT	SBT (CoP velocity↑, CoP velocity in AP direction↓, CoP velocity in ML direction↑)
Sannicandro 2020 (35), Italy	IG: 38 CG: 41	M/F	IG: 68.2 ± 2.1 CG: 68.9 ± 2.2	CT	Walking+Joint mobility+Flexibility (static and dynamic tasks)	10 weeks	Three times a week; 30-45 min	MGT, SLST, WT, TUG, BWT	MGT (sit-up↑, extension↑, side plank right↑, side plank left↑), OLST (right↑, left↑), WT↑, TUG (8 m↑), BWT (3 m↑)
Yoon 2024 (36), Korea	IG: 24 CG: 21	M/F	IG: 78.46 ± 5.12 CG: 78.14 ± 3.9	PT (CT)	PT + Clapping	6 weeks	Twice a week; 40-50 min	DBT (GT, WT), SBT (FRT)	GT (velocity↑, cadence↑, right step time↓, Left step time↓), WT (10 m↑), FRT↑
Koh 2016 (32), Korea	IG: 24	F	IG: 77.87 ± 6.95	CT	N/A	8 weeks	Twice a week; 20-30 min	SPPB (BT, GT, RCS), GUD, SLST	SPPB↑ (BT↓, GT↑, RCS (5 times↑)), GUD (4 stairs↑), OLST (open eyes↑, closed eyes↑)
Choi 2021 (37), Korea	IG: 22	F	IG: ≥ 65	PT (CT)	N/A	10 weeks	Twice a week; 30 min	GT, 30CST	GT (velocity↓, cadence↓, right step length↑, left step length↑, stride length↑), 30CST↑
Carrasco-Poyatos 2019 (38), Spain	IG1: 16 IG2: 19 CG: 14	F	IG/CG: 60–80	PT (CT, IG1) RT (IG2)	No exercise program	18 weeks	Twice a week; 60 min	SBT, DBT	SBT (OLST↑, velocity in ML↑, velocity in AP↑, velocity↑), DBT (TUG↓)
Petrofsky 2005 (12), California	IG: 13	M/F	IG: 73.1 ± 7.3	CT with the 6-s abs machine	N/A	4 weeks	Three times a week; 20 min	FRT	FRT (forward↑, right↑, left↑)
Kang 2012 (40), Korea	IG: 15 CG: 15	M/F	IG: 65–80 CG: 65–80	CT	No training	8 weeks	30 min each session	BBS, Tetrax	BBS↑, Stability↑
Hosseini 2012 (39), Iran	IG1: 30 IG2: 30 CG: 30	M/F	IG1: 65.3 ± 4.8 IG2: 63.7 ± 4.23 CG: 60.76 ± 5.09	ST (IG1) CT (IG2)	No training	6 weeks	Three times a week; 60 min	Y-BT, DGI	Y-BT↑, DGI↑

IG, intervention group; CG, control group; M, male; F, female; CT, core training (core muscle training or core instability strength training or core resistance training OR core stability training); PT, Pilates training; ST, strength Training; Y-BT, Y-balance test; DGI, dynamic gait index; BBS, berg’s balance scale; RT, resistance training; FRT, functional reach test; SEBT, star excursion balance test; DBT, dynamic balance testing; SBT, static balance testing; FM, functional mobility; MGT, McGill test; OLST, one-leg stance test; TUG: timed up and go test; BWT, backwards walk test; GT, gait test; WT, walk test; BT, balance test; SPPB, short physical performance battery; RCS, repeated chair stands; GUD, going up and down; 30CST, 30-s chair sit-to-stand test; CoP, center-of-pressure; ML, medial-lateral; AP, anterior–posterior; ↑, significant within-group improvement; ↓, non-significant within-group.

In addition to these primary assessment tools, several studies employed alternative tests to evaluate various dimensions of balance and physical performance. These included the Star Excursion Balance Test (SEBT), Center-of-Pressure (CoP) test, Backward Walk Test (BWT), Short Physical Performance Battery (SPPB), Repeated Chair Stands (RCS), Going Up and Down (GUD), Strength Training (ST); Y-Balance Test (Y-BT); Dynamic gait index (DGI); Berg’s balance scale (BBS) and the Chair Sit-to-Stand Test (CST).

### 3.3 Risk of bias

**Figure 2:**
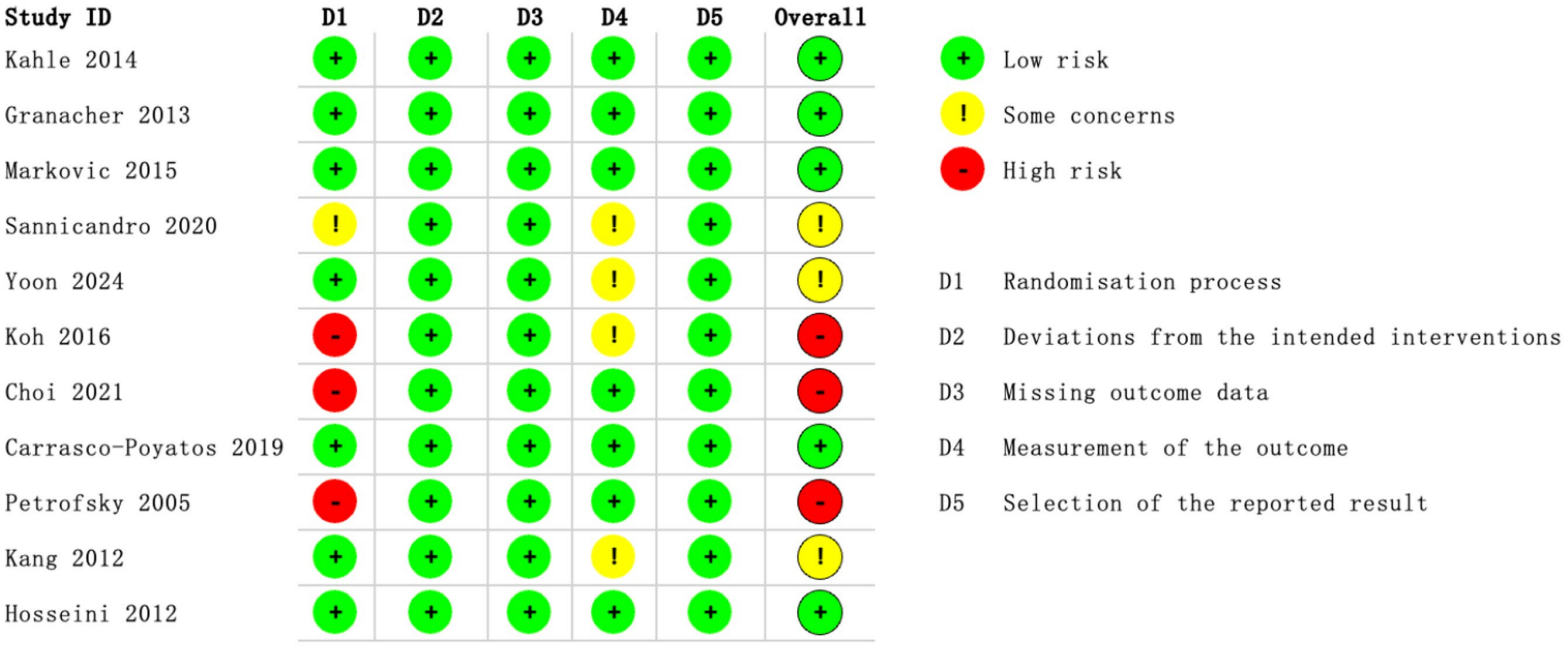
Risk of bias summary: methodological quality of each item for the each included study.

**Figure 3:**
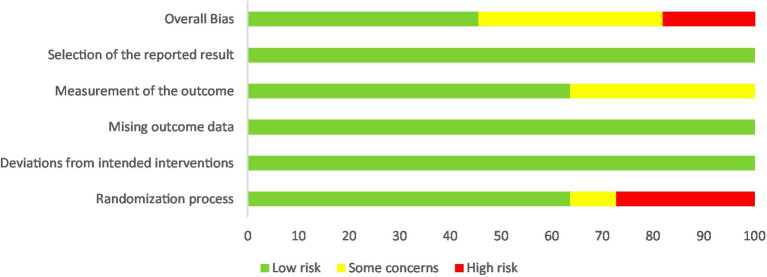
Risk of overall bias.

The risk of bias assessment for individual studies, conducted using the RoB 2 tool, is depicted in Figure 2, while Figure 3 provides an aggregate overview of the overall risk of bias across all included studies. In these visualizations, green indicates low risk, yellow denotes some concerns, and red signifies high risk of bias. Regarding biases in the randomization process, seven studies explicitly reported their randomization methods, ensuring transparency and reliability. In contrast, four studies lacked sufficient details (12, 32, 35, 37), with three of them (12, 32, 37) employing single-group designs, leading to a high-risk rating. Deviations from intended interventions posed unique challenges in exercise training research due to the direct involvement of participants in interventions and the frequent dual role of researchers as outcome assessors. While only three studies (34, 37, 38) explicitly implemented blinding procedures, the lack of such measures in the remaining studies did not appear to influence outcomes. For missing outcome data, all studies were rated as low risk, indicating that the absence of data did not substantially impact the results. However, in the domain of bias related to outcome measurement, four studies (32, 35, 36, 40) did not clearly address whether outcome measurement differed between groups. In terms of selective reporting bias, all studies were assessed as low risk, reflecting adequate reporting practices. Regarding overall bias, five studies (31, 33, 34, 38, 39) demonstrated a low risk of bias. Meanwhile, three studies (35, 36, 40) raised some concerns, and three study (12, 32, 37) was identified as having a high risk of bias.

### 3.4 Meta-analysis results

A total of eleven studies were included in the meta-analysis, focusing on outcome measures related to both dynamic and static balance. For DBT, three specific assessments were examined: the GT, reported in four studies (32, 33, 36, 37); the FRT, included in Four studies (12, 31, 33, 36); and the TUG Test, also evaluated in three studies (33, 35, 38). For SBT, the OLST was analyzed across three studies (32, 35, 38). These outcome measures collectively provide a robust framework for evaluating the effects of CT interventions on balance, encompassing both dynamic and static dimensions, and offering a comprehensive synthesis of evidence across the included studies.

#### 3.4.1 Effect of CT on DBT (GT velocity, FRT distance, and TUG time)

**Figure 4:**
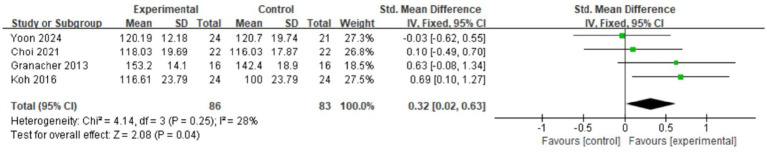
Forest plot of CT on GT.

Four studies (*n* = 169) (32, 33, 36, 37) examined the effect of CT on GT velocity in older adults. As illustrated in the Figure 4, I^2^ = 28%, indicating moderate heterogeneity, and a fixed-effects model was applied. The meta-analysis demonstrated an (SMD = 0.32; 95%CI = 0.02, 0.63; *p* < 0.05). These findings suggest that CT significantly improves GT velocity in older adults, with the experimental group outperforming the control group.

**Figure 5:**
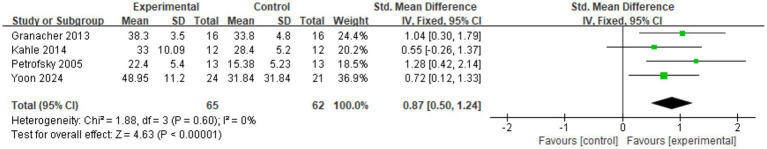
Forest plot of CT on FRT.

Four studies (*n* = 127) (12, 31, 33, 36) investigated the effect of CT on FRT distance in older adults. As shown in the Figure 5, I^2^ = 0%, indicating low heterogeneity, and a fixed-effects model was applied. The meta-analysis yielded an (SMD = 0.82; 95%CI = 0.50, 1.24; *p* < 0.00001). These results indicate that CT significantly improves FRT distance in older adults, with the experimental group demonstrating superior performance compared to the control group.

**Figure 6:**
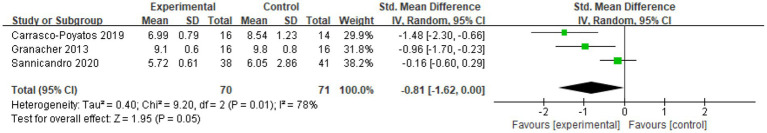
Forest plot of CT on TUG.

Three studies (*n* = 141) (33, 35, 38) explored the effect of CT on TUG time in older adults. As illustrated in the Figure 6, I^2^ = 78%, indicating substantial heterogeneity, and a random-effects model was employed. The meta-analysis revealed an (SMD = −0.81; 95% CI = −1.62, 0.00; *p* = 0.05). These findings suggest that CT significantly improves TUG time in older adults, with the experimental group showing superior performance compared to the control group.

#### 3.4.2 Effect of CT on SBT (OLST time)

**Figure 7:**
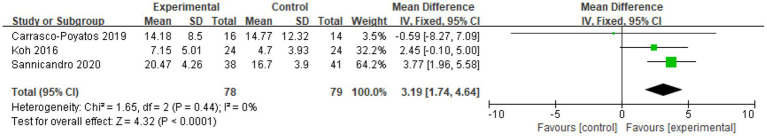
Forest plot of CT on OLST(s).

Three studies (*n* = 157) (32, 35, 38) evaluated the effect of CT on OLST time in older adults. As depicted in the Figure 7, I^2^ = 0%, indicating no heterogeneity, and a fixed-effects model was applied. The meta-analysis showed an (MD = 3.19; 95% CI = 1.74, 4.64; *p* < 0.001). These results demonstrate that CT significantly enhances OLST time in older adults, with the experimental group performing better than the control group.

### 3.5 Subgroup analysis

In this study, subgroup analyses were performed for DBT, the TUG test, and GT to explore the sources of heterogeneity in the outcome measures.

**Figure 8:**
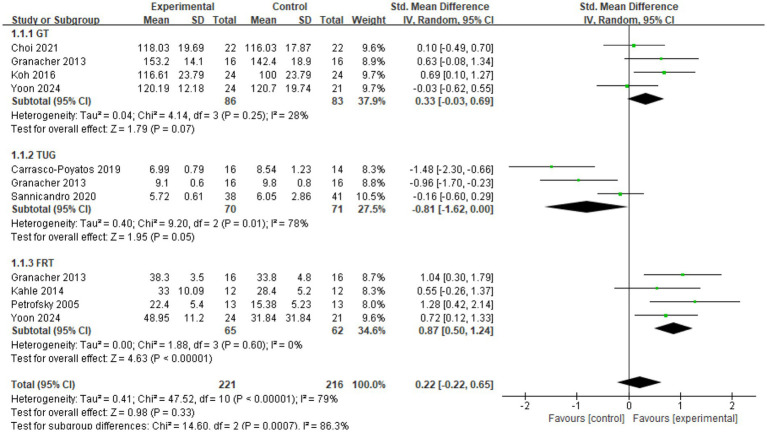
Subgroup analysis of DBT.

The results suggest that the type of DBT assessment method significantly contributes to the observed heterogeneity in DBT outcomes (Figure 8). Specifically, the subgroup using the FRT exhibited a statistically significant effect (SMD = 0.82; 95%CI = 0.50, 1.24; *p* < 0.00001; I^2^ = 0%), as did the TUG subgroup (SMD = −0.81; 95% CI: −1.62, 0.00; *p* = 0.05; I^2^ = 78%). In contrast, the GT subgroup did not demonstrate a statistically significant result (SMD = 0.33; 95% CI: −0.03, 0.69; *p* = 0.07; I^2^ = 28%). These findings indicate that the variability in DBT outcomes is likely due to the differences in the assessment methods used. Notably, CT had the most pronounced effect on FRT performance in older adults, while its impact on TUG and GT was less significant.

**Figure 9:**
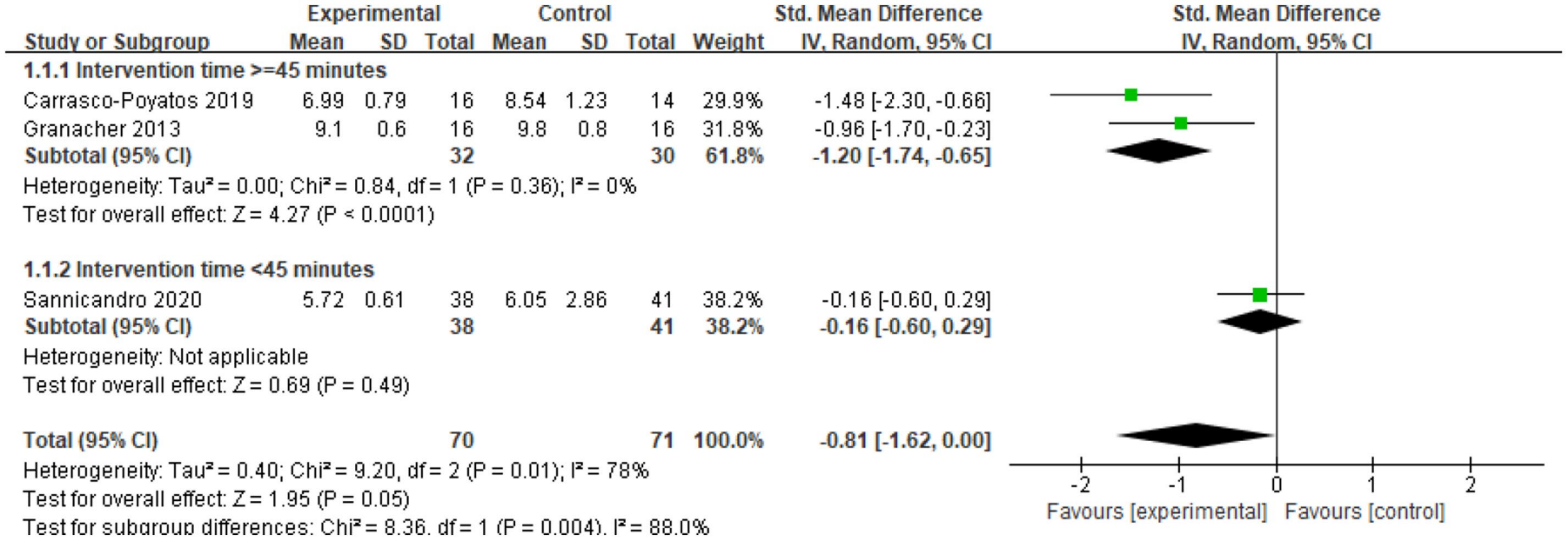
Subgroup analysis of TUG.

The analysis also revealed that intervention duration plays a key role in the heterogeneity observed in the TUG outcome measure (Figure 9). When participation duration was considered, the subgroup with an intervention time of ≥45 min demonstrated a significant effect (SMD = −1.20; 95% CI: −1.74, −0.65; *p* < 0.0001; I^2^ = 0%). This choice of 45 min was not arbitrary; earlier meta-analyses on balance training, as well as reviews of Tai Chi and Pilates, have repeatedly demonstrated that programs with sessions of at least this length are more likely to produce meaningful improvements in postural stability among older adults (19, 23, 41). Drawing on this body of evidence, we applied 45 min as a practical and evidence-based threshold for subgrouping in the present study. However, the subgroup with an intervention time of <45 min did not show statistically significant results (SMD = −0.16; 95% CI: −0.60, 0.29; *p* = 0.49). The overall effect size was calculated as (SMD = −0.81; 95% CI: −1.62, 0.00; *p* = 0.05). These findings underscore the importance of intervention duration in the variability of TUG outcomes. The subgroup with a longer intervention duration (≥45 min) exhibited a significantly higher effect size, suggesting that extended training periods have a more substantial impact on TUG performance. This highlights the advantages of longer training sessions over shorter ones in improving balance through CT, particularly for older adults.

**Figure 10:**
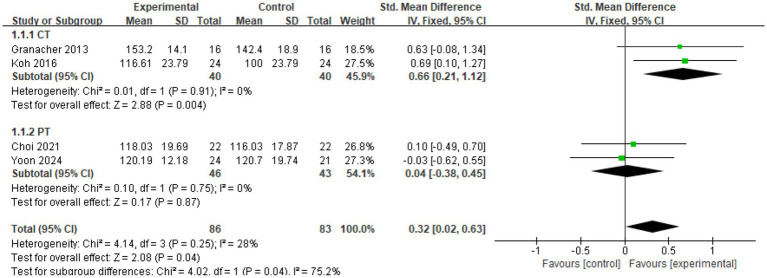
Subgroup analysis of GT.

Finally, the analysis of intervention types revealed that different approaches to intervention contribute significantly to the heterogeneity observed in GT outcomes (Figure 10). The CT subgroup demonstrated a statistically significant effect (SMD = 0.66; 95% CI: 0.21, 1.12; *p* < 0.005; I^2^ = 0%), whereas the PT subgroup did not show statistically significant results (SMD = 0.04; 95% CI: −0.38, 0.45; *p* > 0.05; I^2^ = 0%). The overall effect size was calculated as (SMD = 0.32; 95% CI: 0.02, 0.63; *p* < 0.05). These findings suggest that the variation in GT outcomes is attributable to the type of intervention used. Importantly, the effectiveness of CT was substantially greater than that of PT, highlighting the stronger impact of CT interventions on GT performance in older adults.

### 3.6 Publication bias test

In this study, given the limited number of articles, publication bias was assessed using Begg’s rank correlation method and Egger’s linear regression method. A *p*-value exceeding 0.05 suggests minimal publication bias, whereas values below this threshold indicate substantial bias. The trim and fill method, which assesses and corrects for publication bias based on asymmetry in funnel plots, was employed to mitigate the impact of bias on the meta-analysis results. This method involves adding “missing” studies to the funnel plot to balance the graphic representation, thereby reducing the influence of publication bias.

Table 3 presents the publication bias assessment for this research. For GT, FRT, OLST, and DBT, both Begg’s and Egger’s test values were above 0.05, suggesting little evidence of publication bias. However, for the TUG outcome, Egger’s test was significant (*p* = 0.01), while Begg’s test remained non-significant (*p* = 0.30), indicating a potential risk of bias. Subsequent application of the trim-and-fill method revealed that Hedges’s g shifted from −0.81 (95% CI: −1.59, −0.03) to −0.16 (95% CI, −1.10, 0.79) after imputing missing studies (Table 4). This adjustment substantially attenuates the effect size, suggesting that the observed benefit on TUG performance may have been overestimated and should therefore be interpreted with caution.

**Table 3:** Publication Bias Test.

Study	Egger’s test	Begg’s test
GT	0.37	1.00
FRT	0.55	0.73
TUG	0.01	0.30
OLST	0.22	0.30
DBT	0.92	0.53

**Table 4:** Trim and fill method.

Study		Hedges’s g	[95% Confidence interval]	
TUG	Observed	−0.81	−1.59	−0.03
	Observed + Imputed	−0.16	−1.10	0.79

## 4 Discussion

### 4.1 Study findings

This study conducted a systematic review and meta-analysis to evaluate the effects of CT on balance performance in older adults. The findings demonstrate a significant positive impact of CT on balance, particularly in both dynamic and static balance assessments. Specifically, CT not only yielded beneficial effects in the short term, but these improvements were consistently observed across multiple balance measures. The results indicate that CT significantly enhanced balance in older adults, particularly in dynamic balance assessments such as the FRT, the TUG test, and GT, as well as in static balance assessments like the OLST. These findings highlight the crucial role of CT in enhancing multidimensional balance abilities.

### 4.2 The impact of CT on balance performance in older adults

Subgroup analyses revealed that CT had the most pronounced effect on dynamic balance in the FRT. This outcome is likely attributable to the critical role of core muscles in controlling the center of mass and maintaining trunk stability. The FRT requires older adults to manage their center of mass while leaning forward, and CT enhances the strength and stability of the core muscles, thereby significantly improving performance in this dynamic balance task. In contrast, the effects of CT were less pronounced in the GT and the TUG test. For the TUG outcome, the results warrant cautious interpretation. Egger’s test indicated significant funnel asymmetry (*p* = 0.01), and when the trim-and-fill method was applied, the pooled effect declined from −0.81 to −0.16, with the confidence interval spanning zero (Tables 3, 4). These findings suggest that, once potential missing studies are considered, the benefit of CT for TUG is less certain. It is also worth noting that gait control depends not only on core stability but also on lower-limb strength and motor coordination, which may help explain the comparatively modest effects of CT on GT and TUG.

Further analysis showed that training sessions lasting more than 45 min yielded better results in dynamic balance. This finding may be linked to the relationship between training duration and neuromuscular adaptation. Longer sessions provide participants with more opportunities for repeated practice, which not only strengthens the core muscles but also enhances the nervous system’s ability to coordinate and remember movement patterns (42). For older adults, extended training times contribute to improved neuromuscular coordination, enabling more precise control over body movements, thereby enhancing overall balance. Conversely, shorter training sessions may not allow for sufficient neuromuscular adaptation. CT requires prolonged stimulation to activate muscle groups and induce adaptive changes and short sessions may only lead to basic strength gains without improving complex dynamic balance capabilities (10). Extending the training duration enhances not only core muscle strength but also the body’s ability to adapt to a variety of movement challenges, which is especially critical for dynamic balance. Dynamic balance requires maintaining stability in an ever-changing environment, and this ability is often best achieved through sustained, long hours of training.

In terms of different intervention types, CT showed superior effects on GT compared to PT. This result is theoretically supported, as gait performance is closely related to dynamic body control, particularly in terms of step coordination, stride length, and gait symmetry. CT enhances the stability of the trunk, hips, abdomen, and back muscles, which in turn improves the stability of these regions during gait. A stable core effectively regulates lower limb movement, preventing unnecessary energy expenditure and unbalanced movement patterns, thus enhancing gait efficiency and stability. While Pilates also emphasizes core strength, its focus on flexibility, breath control, and body awareness may be less effective than CT in improving core strength and stability.

In comparison to the existing literature, the results of this study corroborate several previous findings. Previous research (43) similarly reported significant improvements in balance following CT, particularly in dynamic balance tasks. Strengthening the core musculature enables older adults to markedly enhance their balance control, a conclusion that closely aligns with our findings. Moreover, this study is the first to perform a systematic review across diverse cultural contexts, thus broadening the scope of the existing literature. While similar results were reported in Wang Hangping’s study (262,019), which primarily focused on a specific regional and age group, our research, by synthesizing data from various geographical and demographic groups, offers more generalized conclusions, further substantiating the positive effects of CT on balance in older adults.

### 4.3 The potential physiological mechanisms of CT on balance performance in the older adult

From a mechanistic perspective, CT effectively strengthens the core musculature, particularly the abdominal and lumbar muscles, which are essential for overall body stability and postural control (44). By enhancing core strength, older adults become better equipped to maintain stable postures, especially during standing or walking, thereby reducing the risk of falls. The beneficial effects on balance may be attributed to improvements in self-support capacity, postural adjustments, and coordination across various body segments. From a neurological standpoint, long-term engagement in such training not only increases muscle strength but also optimizes the adaptive capacity of the nervous system, thereby enhancing control over movement tasks in older adults (42). Studies in older adults have provided direct evidence that CT induces measurable physiological changes, such as hypertrophy of trunk muscles, improvements in isometric trunk strength, and enhanced neuromuscular control, thereby lending empirical support to these proposed mechanisms (33). Strengthened core musculature improves the feedback mechanisms within the nervous system that regulate postural adjustments, enabling older individuals to stabilize their balance rapidly in unstable conditions, contributing to enhanced dynamic balance performance. Furthermore, core-focused exercises may enhance proprioception, enabling quicker responses to instability (45). This mechanism is critical for both static and dynamic balance assessments and is particularly valuable in daily activities, where this type of training significantly enhances stability and independence.

This study not only provides empirical evidence for the effects of CT on balance in older adults but also contributes to the advancement of exercise physiology and training theory. Much of the existing literature has predominantly focused on the effects of CT in younger athletes (46, 47), whereas this study expands the scope by examining its unique benefits for the older population. Furthermore, this research provides new evidence supporting the “functional training theory,” which posits that exercise interventions should emphasize enhancing functional abilities relevant to daily life, such as balance, stability, and coordination (48). Our findings demonstrate that CT significantly improves balance in older adults, particularly dynamic balance, thereby validating the functional training theory and bolstering its practical application in promoting older adult health.

### 4.4 The potential of CT for practical enhancement in older adult care

From a practical perspective, CT has been shown to significantly improve balance, particularly dynamic balance. As such, it should be adopted as a standard intervention to enhance stability and prevent falls among older adults. Policymakers can utilize the findings of this study to integrate CT into health promotion initiatives targeting older populations, thereby facilitating improvements in their overall quality of life. Beyond its role in improving balance, this intervention also enhances independence in daily activities. Given the mobility challenges and heightened fall risk faced by this demographic, CT empowers older adults to perform routine tasks with greater confidence. Consequently, educators and community service organizations should actively advocate for its implementation as a critical component of programs designed to promote health and quality of life in the older adult.

### 4.5 Limitation and future research

While this study contributes to the understanding of CT and balance in older adults, several limitations should be considered when interpreting the findings. A small number of the included trials adopted single-group designs, which may reduce the strength of causal inferences. Although strict data extraction procedures were applied, the possibility of publication bias cannot be excluded. Results from the trim-and-fill analysis suggested that this bias may have influenced the pooled estimates to some extent, though the overall conclusions appear relatively stable.

Although heterogeneity was systematically examined and prespecified subgroup analyses were conducted, the limited number of included studies precluded meta-regression, which restricts the ability to quantify moderator effects more precisely. Moreover, the relatively small number of available trials means that some results should be interpreted with caution, as the restricted evidence base may reduce the stability and generalizability of the findings.

The methodological quality of the included studies also reflects the typical challenges of exercise intervention research. Blinding procedures were often difficult to implement, and sample sizes tended to be modest, which may limit statistical precision and increase the likelihood of small-study effects. Furthermore, the evidence base is weighted toward short-term interventions, offering limited information on whether the benefits of CT can be maintained over longer periods or translated into meaningful reductions in fall risk.

Variation in intervention protocols represents another limitation. Differences in training intensity, session length, adherence monitoring, and reporting practices complicate direct comparisons across studies and make it difficult to determine optimal training parameters. Regarding subgroup analyses, the 45-min threshold had already been justified earlier on the basis of prior research, and in this context was applied pragmatically to distinguish between shorter and longer training sessions. Nonetheless, residual heterogeneity is likely shaped by factors beyond duration and intervention type, including participants’ baseline balance capacity, intervention settings, adherence levels, and even differences in outcome measurement procedures, such as walkway length in the TUG or arm positioning in the OLST. In addition, some subgroup comparisons were based on relatively few studies, which restricts the precision of estimates and raises the possibility of small-study effects.

Finally, cultural and geographical concentration of the included studies may limit the broader applicability of the findings, as exercise behaviors and responses are often shaped by social norms, healthcare systems, and lifestyle contexts.

Taken together, these limitations highlight the need for future trials to recruit larger and more diverse samples, implement standardized intervention protocols, and extend follow-up durations to evaluate the long-term sustainability of training effects. Expanding research across varied cultural contexts would further enhance the generalizability of findings and provide a more comprehensive picture of how CT contributes to balance and fall prevention in older adults.

## 5 Conclusion

This systematic review and meta-analysis provides an in-depth examination of the effects of CT on balance performance in older adults. The findings demonstrate significant improvements across dynamic and static balance measures, including GT, FRT, TUG and OLST. Nevertheless, the TUG results should be interpreted with caution. These results highlight the efficacy of CT as a targeted intervention for enhancing balance and reducing fall risk in this vulnerable population. This study contributes to the growing body of literature advocating CT as a crucial component in improving health outcomes for older adults. The findings hold particular relevance for clinicians and practitioners designing rehabilitation programs, especially those focused on promoting balance and fall prevention.

## Data Availability

The datasets presented in this study can be found in online repositories. The names of the repository/repositories and accession number(s) can be found in the article/Supplementary material.
