# Supplementary material for: Effects of core training on balance performance in older adults: a systematic review and meta-analysis
Source: Front Public Health. 2025 Oct 9;13:1661460. doi: 10.3389/fpubh.2025.1661460 (PMC12548199; doi:10.3389/fpubh.2025.1661460)
Supplement: Supplementary file 1 [file Data_Sheet_1.zip › Supplementary Materials/Table S1.docx]

**Table S1.** The complete search strategy for the databases

| **Database** | **Complete Search Strategy** |
| --- | --- |
| PubMed | ((((((Core Training[MeSH]) OR (Core Strength Training[Title/Abstract])) OR (Core-Muscle Training[Title/Abstract])) OR (Core-Stability Exercise[Title/Abstract])) OR (Core Exercise[Title/Abstract])) AND (((((balance[Title/Abstract]) OR (static balance[Title/Abstract])) OR (dynamic balance[Title/Abstract])) OR (functional balance[Title/Abstract])) OR (postural balance[MeSH]))) AND ((((aged[MeSH]) OR (old[Title/Abstract])) OR (senior[Title/Abstract])) OR (elder[Title/Abstract])) |
| Cochrane Library | #1 MeSH descriptor: [Postual Balance] explode all trees |
|  | #2 (balance):ti,ab,kw OR (static balance):ti,ab,kw OR (dynamic balance):ti,ab,kw OR (functional balance):ti,ab,kw |
|  | #3 #1 OR #2 |
|  | #4 MeSH descriptor: [Aged] in all MeSH products |
|  | #5 (old):ti,ab,kw OR (senior):ti,ab,kw OR (elder):ti,ab,kw |
|  | #6 #4 OR #5 |
|  | #7 (Core training):ti,ab,kw OR (Core Strength Training):ti,ab,kw OR (Core-Muscle Training):ti,ab,kw OR (Core-Stability Exercise):ti,ab,kw OR (Core Exercise):ti,ab,kw |
|  | #8 #3 AND #6 AND #7 |
| Embase | #1 'aged'/exp |
|  | #2 'old':ab,ti OR 'senior':ab,ti OR 'elder':ab,ti |
|  | #3 'core strength training':ab,ti OR 'core-muscle training':ab,ti OR 'core training':ab,ti OR 'core-stability exercise':ab,ti OR 'core exercise':ab,ti |
|  | #4 'balance':ab,ti OR 'static balance':ab,ti OR 'dynamic balance':ab,ti OR 'functional balance':ab,ti OR 'postural balance':ab,ti |
|  | #5 #1 OR #2 |
|  | #6 #3 AND #4 AND #5 |
| Web of Science | ((((TS=(balance)) OR TS=(static balance)) OR TS=(dynamic balance)) OR TS=(functional balance)) OR TS=(postural balance) AND ((((TS=(Core Strength Training)) OR TS=(Core-Muscle Training)) OR TS=(Core training)) OR TS=(Core-Stability Exercise)) OR TS=(Core Exercise) AND (((TS=(old)) OR TS=(aged)) OR TS=(senior)) OR TS=(elder) |
| EBSCOhost | S1 AB Core Strength Training OR AB Core-Muscle Training OR AB Core training OR AB Core-Stability Exercise OR AB Core Exercise |
|  | S2 AB balance OR AB static balance OR AB dynamic balance OR AB functional balance OR AB postural balance |
|  | S3 AB old OR AB aged OR AB senior OR AB elder |
|  | S4 S1 AND S2 AND S3 |
