# Supplementary material for: Effects of core training on balance performance in older adults: a systematic review and meta-analysis
Source: Front Public Health. 2025 Oct 9;13:1661460. doi: 10.3389/fpubh.2025.1661460 (PMC12548199; doi:10.3389/fpubh.2025.1661460)
Supplement: Supplementary file 1 [file Data_Sheet_1.zip › Supplementary Materials/Supplementary Materials A.pdf]

Unique ID	Kahle 2014	Study ID	Kahle 2014	Assessor	Guo
Ref or Label	Journal of Aging and Physical Activity, 2014,22, 65-73	Aim	assignment to intervention (the 'intention-to-treat' effect)		
Experimental	CT	Comparator	Routine training	Source	Journal article(s)
Outcome	FRT	Results	Cohen's d=1.30	Weight	1

Domain	Signalling question	Response	Comments
Bias arising from the randomization process	1.1 Was the allocation sequence random?	Y	Participants were randomly assigned to either and exercise or control group in a 1–1 ratio. The randomization scheme was generated using the Web site Randomization. com. Allocation was
	1.2 Was the allocation sequence concealed until participants were enrolled and assigned to interventions?	Y	
	1.3 Did baseline differences between intervention groups suggest a problem with the randomization process?	N	There were no significant differences between the control and exercise group for [illegible]
	**Risk of bias judgement**	**Low**	
Bias due to deviations from intended interventions	2.1.Were participants aware of their assigned intervention during the trial?	Y	participants underwent diffierent procedures that cannot be blinded
	2.2.Were carers and people delivering the interventions aware of participants' assigned intervention during the trial?	Y	
	2.3. If Y/PY/NI to 2.1 or 2.2: Were there deviations from the intended intervention that arose because of the experimental context?	N	
	2.4 If Y/PY to 2.3: Were these deviations likely to have affected the outcome?	NA	
	2.5. If Y/PY/NI to 2.4: Were these deviations from intended intervention balanced between groups?	NA	
	2.6 Was an appropriate analysis used to estimate the effect of assignment to intervention?	Y	
	2.7 If N/PN/NI to 2.6: Was there potential for a substantial impact (on the result) of the failure to analyse participants in the group to which they were randomized?	NA	
	**Risk of bias judgement**	**Low**	
Bias due to missing outcome data	3.1 Were data for this outcome available for all, or nearly all, participants randomized?	Y	The proporting of missing follow-up was 2/26
	3.2 If N/PN/NI to 3.1: Is there evidence that result was not biased by missing outcome data?	NA	
	3.3 If N/PN to 3.2: Could missingness in the outcome depend on its true value?	NA	
	3.4 If Y/PY/NI to 3.3: Is it likely that missingness in the outcome depended on its true value?	NA	
	**Risk of bias judgement**	**Low**	
Bias in measurement of the outcome	4.1 Was the method of measuring the outcome inappropriate?	N	
	4.2 Could measurement or ascertainment of the outcome have differed between intervention groups?	PN	
	4.3 Were outcome assessors aware of the intervention received by study participants?	Y	
	4.4 If Y/PY/NI to 4.3: Could assessment of the outcome have been influenced by knowledge of intervention received?	N	
	4.5 If Y/PY/NI to 4.4: Is it likely that assessment of the outcome was influenced by knowledge of intervention received?	NA	
	**Risk of bias judgement**	**Low**	
Bias in selection of the reported result	5.1 Were the data that produced this result analysed in accordance with a pre-specified analysis plan that was finalized before unblinded outcome data were available for analysis?	Y	
	5.2 ... multiple eligible outcome measurements (e.g. scales, definitions, time points) within the outcome domain?	N	
	5.3 ... multiple eligible analyses of the data?	N	
	**Risk of bias judgement**	**Low**	
Overall bias	**Risk of bias judgement**	**Low**	

Unique ID	Granacher 2013	Study ID	Granacher 2013	Assessor	Guo
Ref or Label	Gerontology 2013;59:105–113	Aim	assignment to intervention (the 'intention-to-treat' effect)		
Experimental	CT	Comparator	Routine training	Source	Journal article(s)
Outcome	GT,FRT,TUG	Results	f = 0.41 and 0.59	Weight	1

Domain	Signalling question	Response	Comments
Bias arising from the randomization process	1.1 Was the allocation sequence random?	Y	Participants were randomly assigned into an intervention (INT) group and a control (CON) group. The randomization process was done using Research Randomizer, a program published on a publicly accessible official
	1.2 Was the allocation sequence concealed until participants were enrolled and assigned to interventions?	Y	
	1.3 Did baseline differences between intervention groups suggest a problem with the randomization process?	N	At baseline, all subjects met the inclusion criteria (e.g., MMSE, CDT) for participating in [illegible]
	**Risk of bias judgement**	**Low**	
Bias due to deviations from intended interventions	2.1.Were participants aware of their assigned intervention during the trial?	PY	participants underwent diffierent procedures that cannot be blinded
	2.2.Were carers and people delivering the interventions aware of participants' assigned intervention during the trial?	PY	
	2.3. If Y/PY/NI to 2.1 or 2.2: Were there deviations from the intended intervention that arose because of the experimental context?	N	
	2.4 If Y/PY to 2.3: Were these deviations likely to have affected the outcome?	NA	
	2.5. If Y/PY/NI to 2.4: Were these deviations from intended intervention balanced between groups?	NA	
	2.6 Was an appropriate analysis used to estimate the effect of assignment to intervention?	PY	

	2.7 If N/PN/NI to 2.6: Was there potential for a substantial impact (on the result) of the failure to analyse participants in the group to which they were randomized?	NA	
	**Risk of bias judgement**	**Low**	
**Bias due to missing outcome data**	3.1 Were data for this outcome available for all, or nearly all, participants randomized?	NI	
	3.2 If N/PN/NI to 3.1: Is there evidence that result was not biased by missing outcome data?	N	
	3.3 If N/PN to 3.2: Could missingness in the outcome depend on its true value?	N	
	3.4 If Y/PY/NI to 3.3: Is it likely that missingness in the outcome depended on its true value?	NA	
	**Risk of bias judgement**	**Low**	
**Bias in measurement of the outcome**	4.1 Was the method of measuring the outcome inappropriate?	N	
	4.2 Could measurement or ascertainment of the outcome have differed between intervention groups?	PN	
	4.3 Were outcome assessors aware of the intervention received by study participants?	NI	
	4.4 If Y/PY/NI to 4.3: Could assessment of the outcome have been influenced by knowledge of intervention received?	N	
	4.5 If Y/PY/NI to 4.4: Is it likely that assessment of the outcome was influenced by knowledge of intervention received?	NA	
	**Risk of bias judgement**	**Low**	
**Bias in selection of the reported result**	5.1 Were the data that produced this result analysed in accordance with a pre-specified analysis plan that was finalized before unblinded outcome data were available for analysis?	Y	
	5.2 ... multiple eligible outcome measurements (e.g. scales, definitions, time points) within the outcome domain?	N	
	5.3 ... multiple eligible analyses of the data?	N	
	**Risk of bias judgement**	**Low**	
**Overall bias**	**Risk of bias judgement**	**Low**	

**Unique ID**	Markovic 2015	**Study ID**	Markovic 2015	**Assessor**	Guo
**Ref or Label**	Archives of Gerontology and Geriatrics	**Aim**	assignment to intervention (the 'intention-to-treat' effect)		
**Experimental**	CT	**Comparator**	PT	**Source**	Journal article(s)
**Outcome**	SBT	**Results**		**Weight**	1

Domain	Signalling question	Response	Comments
**Bias arising from the randomization process**	1.1 Was the allocation sequence random?	Y	[illegible] criteria and were randomly divided into a Pilates and a Huber group. The group allocation schedule was developed by a [illegible]
	1.2 Was the allocation sequence concealed until participants were enrolled and assigned to interventions?	Y	
	1.3 Did baseline differences between intervention groups suggest a problem with the randomization process?	N	
	**Risk of bias judgement**	**Low**	
**Bias due to deviations from intended interventions**	2.1.Were participants aware of their assigned intervention during the trial?	PY	The investigator that performed pre- and posttreatment outcome measurements remained blind to group allocation.
	2.2.Were carers and people delivering the interventions aware of participants' assigned intervention during the trial?	N	
	2.3. If Y/PY/NI to 2.1 or 2.2: Were there deviations from the intended intervention that arose because of the experimental context?	N	
	2.4 If Y/PY to 2.3: Were these deviations likely to have affected the outcome?	NA	
	2.5. If Y/PY/NI to 2.4: Were these deviations from intended intervention balanced between groups?	NA	
	2.6 Was an appropriate analysis used to estimate the effect of assignment to intervention?	Y	
	2.7 If N/PN/NI to 2.6: Was there potential for a substantial impact (on the result) of the failure to analyse participants in the group to which they were randomized?	NA	
	**Risk of bias judgement**	**Low**	
**Bias due to missing outcome data**	3.1 Were data for this outcome available for all, or nearly all, participants randomized?	Y	The proporting of missing follow-up was 4/34
	3.2 If N/PN/NI to 3.1: Is there evidence that result was not biased by missing outcome data?	NA	
	3.3 If N/PN to 3.2: Could missingness in the outcome depend on its true value?	NA	
	3.4 If Y/PY/NI to 3.3: Is it likely that missingness in the outcome depended on its true value?	NA	
	**Risk of bias judgement**	**Low**	
**Bias in measurement of the outcome**	4.1 Was the method of measuring the outcome inappropriate?	N	
	4.2 Could measurement or ascertainment of the outcome have differed between intervention groups?	N	
	4.3 Were outcome assessors aware of the intervention received by study participants?	N	
	4.4 If Y/PY/NI to 4.3: Could assessment of the outcome have been influenced by knowledge of intervention received?	NA	
	4.5 If Y/PY/NI to 4.4: Is it likely that assessment of the outcome was influenced by knowledge of intervention received?	NA	
	**Risk of bias judgement**	**Low**	
**Bias in selection of**	5.1 Were the data that produced this result analysed in accordance with a pre-specified analysis plan that was finalized before unblinded outcome data were available for analysis?	Y	
	5.2 ... multiple eligible outcome measurements (e.g. scales, definitions, time points) within the outcome domain?	N	

**the reported result**	5.3 ... multiple eligible analyses of the data?	N	
	**Risk of bias judgement**	**Low**	
**Overall bias**	**Risk of bias judgement**	**Low**	

**Unique ID**	Sannicandro 2020	**Study ID**	Sannicandro 2020	**Assessor**	Guo
**Ref or Label**	Advances in Physical Education, 2020, 10, 421-435	**Aim**	assignment to intervention (the 'intention-to-treat' effect)		
**Experimental**	CT	**Comparator**	static and dynamic tasks	**Source**	Journal article(s)
**Outcome**	OLST,TUG	**Results**		**Weight**	1

Domain	Signalling question	Response	Comments
**Bias arising from the randomization process**	1.1 Was the allocation sequence random?	Y	The study was conducted with active adults (n = 84, 38 males, 46 females) randomly divided into 2 groups
	1.2 Was the allocation sequence concealed until participants were enrolled and assigned to interventions?	NI	
	1.3 Did baseline differences between intervention groups suggest a problem with the randomization process?	NI	
	**Risk of bias judgement**	**Some concerns**	
**Bias due to deviations from intended interventions**	2.1.Were participants aware of their assigned intervention during the trial?	NI	
	2.2.Were carers and people delivering the interventions aware of participants' assigned intervention during the trial?	Y	
	2.3. If Y/PY/NI to 2.1 or 2.2: Were there deviations from the intended intervention that arose because of the experimental context?	N	
	2.4 If Y/PY to 2.3: Were these deviations likely to have affected the outcome?	NA	
	2.5. If Y/PY/NI to 2.4: Were these deviations from intended intervention balanced between groups?	NA	
	2.6 Was an appropriate analysis used to estimate the effect of assignment to intervention?	Y	
	2.7 If N/PN/NI to 2.6: Was there potential for a substantial impact (on the result) of the failure to analyse participants in the group to which they were randomized?	NA	
	**Risk of bias judgement**	**Low**	
**Bias due to missing outcome data**	3.1 Were data for this outcome available for all, or nearly all, participants randomized?	Y	The proporting of missing follow-up was 5/84
	3.2 If N/PN/NI to 3.1: Is there evidence that result was not biased by missing outcome data?	NA	
	3.3 If N/PN to 3.2: Could missingness in the outcome depend on its true value?	NA	
	3.4 If Y/PY/NI to 3.3: Is it likely that missingness in the outcome depended on its true value?	NA	
	**Risk of bias judgement**	**Low**	
**Bias in measurement of the outcome**	4.1 Was the method of measuring the outcome inappropriate?	N	
	4.2 Could measurement or ascertainment of the outcome have differed between intervention groups?	NI	
	4.3 Were outcome assessors aware of the intervention received by study participants?	Y	
	4.4 If Y/PY/NI to 4.3: Could assessment of the outcome have been influenced by knowledge of intervention received?	N	
	4.5 If Y/PY/NI to 4.4: Is it likely that assessment of the outcome was influenced by knowledge of intervention received?	NA	
	**Risk of bias judgement**	**Some concerns**	
**Bias in selection of the reported result**	5.1 Were the data that produced this result analysed in accordance with a pre-specified analysis plan that was finalized before unblinded outcome data were available for analysis?	Y	
	5.2 ... multiple eligible outcome measurements (e.g. scales, definitions, time points) within the outcome domain?	N	
	5.3 ... multiple eligible analyses of the data?	N	
	**Risk of bias judgement**	**Low**	
**Overall bias**	**Risk of bias judgement**	**Some concerns**	

**Unique ID**	Yoon 2024	**Study ID**	Yoon 2024	**Assessor**	Guo
**Ref or Label**	Med Sci Monit, 2024; 30: e945212	**Aim**	assignment to intervention (the 'intention-to-treat' effect)		
**Experimental**	PT	**Comparator**	ROM + Clapping	**Source**	Journal article(s)
**Outcome**	GT,WT,FRT	**Results**		**Weight**	1

Domain	Signalling question	Response	Comments
**Bias arising from the randomization process**	1.1 Was the allocation sequence random?	Y	To perform the randomization procedure for each group of subjects, the subjects were divided by random sampling using random numbers generated by a computer program (Microsoft Co., Redmond, WA, USA) A total
	1.2 Was the allocation sequence concealed until participants were enrolled and assigned to interventions?	Y	
	1.3 Did baseline differences between intervention groups suggest a problem with the randomization process?	N	Age, height, and weight were compared between the 2 groups, but no significant differences were observed
	**Risk of bias judgement**	**Low**	
	2.1.Were participants aware of their assigned intervention during the trial?	NI	

**Bias due to deviations from intended interventions**	2.2.Were carers and people delivering the interventions aware of participants' assigned intervention during the trial?	NI	
	2.3. If Y/PY/NI to 2.1 or 2.2: Were there deviations from the intended intervention that arose because of the experimental context?	N	
	2.4 If Y/PY to 2.3: Were these deviations likely to have affected the outcome?	NA	
	2.5. If Y/PY/NI to 2.4: Were these deviations from intended intervention balanced between groups?	NA	
	2.6 Was an appropriate analysis used to estimate the effect of assignment to intervention?	Y	
	2.7 If N/PN/NI to 2.6: Was there potential for a substantial impact (on the result) of the failure to analyse participants in the group to which they were randomized?	NA	
	**Risk of bias judgement**	**Low**	
**Bias due to missing outcome data**	3.1 Were data for this outcome available for all, or nearly all, participants randomized?	N	The proporting of missing follow-up was 15/60
	3.2 If N/PN/NI to 3.1: Is there evidence that result was not biased by missing outcome data?	N	
	3.3 If N/PN to 3.2: Could missingness in the outcome depend on its true value?	N	
	3.4 If Y/PY/NI to 3.3: Is it likely that missingness in the outcome depended on its true value?	NA	
	**Risk of bias judgement**	**Low**	
**Bias in measurement of the outcome**	4.1 Was the method of measuring the outcome inappropriate?	N	
	4.2 Could measurement or ascertainment of the outcome have differed between intervention groups?	NI	
	4.3 Were outcome assessors aware of the intervention received by study participants?	NI	
	4.4 If Y/PY/NI to 4.3: Could assessment of the outcome have been influenced by knowledge of intervention received?	PN	
	4.5 If Y/PY/NI to 4.4: Is it likely that assessment of the outcome was influenced by knowledge of intervention received?	NA	
	**Risk of bias judgement**	**Some concerns**	
**Bias in selection of the reported result**	5.1 Were the data that produced this result analysed in accordance with a pre-specified analysis plan that was finalized before unblinded outcome data were available for analysis?	Y	
	5.2 ... multiple eligible outcome measurements (e.g. scales, definitions, time points) within the outcome domain?	N	
	5.3 ... multiple eligible analyses of the data?	N	
	**Risk of bias judgement**	**Low**	
**Overall bias**	**Risk of bias judgement**	**Some concerns**	

**Unique ID**	Koh 2016	**Study ID**	Koh 2016	**Assessor**	Guo
**Ref or Label**	Korean Journal of Sport Biomechanics 2016; 26(2): 229-236	**Aim**	assignment to intervention (the 'intention-to-treat' effect)		
**Experimental**	CT	**Comparator**	N/A	**Source**	Journal article(s)
**Outcome**	SPPB,GUD,OLST	**Results**		**Weight**	1

Domain	Signalling question	Response	Comments
**Bias arising from the randomization process**	1.1 Was the allocation sequence random?	N	
	1.2 Was the allocation sequence concealed until participants were enrolled and assigned to interventions?	N	
	1.3 Did baseline differences between intervention groups suggest a problem with the randomization process?	NI	
	**Risk of bias judgement**	**High**	
**Bias due to deviations from intended interventions**	2.1.Were participants aware of their assigned intervention during the trial?	NI	
	2.2.Were carers and people delivering the interventions aware of participants' assigned intervention during the trial?	NI	
	2.3. If Y/PY/NI to 2.1 or 2.2: Were there deviations from the intended intervention that arose because of the experimental context?	N	
	2.4 If Y/PY to 2.3: Were these deviations likely to have affected the outcome?	NA	
	2.5. If Y/PY/NI to 2.4: Were these deviations from intended intervention balanced between groups?	NA	
	2.6 Was an appropriate analysis used to estimate the effect of assignment to intervention?	Y	
	2.7 If N/PN/NI to 2.6: Was there potential for a substantial impact (on the result) of the failure to analyse participants in the group to which they were randomized?	NA	
	**Risk of bias judgement**	**Low**	
**Bias due to missing outcome data**	3.1 Were data for this outcome available for all, or nearly all, participants randomized?	NI	
	3.2 If N/PN/NI to 3.1: Is there evidence that result was not biased by missing outcome data?	N	
	3.3 If N/PN to 3.2: Could missingness in the outcome depend on its true value?	N	
	3.4 If Y/PY/NI to 3.3: Is it likely that missingness in the outcome depended on its true value?	NA	
	**Risk of bias judgement**	**Low**	
**Bias in measurement of**	4.1 Was the method of measuring the outcome inappropriate?	N	
	4.2 Could measurement or ascertainment of the outcome have differed between intervention groups?	NI	
	4.3 Were outcome assessors aware of the intervention received by study participants?	NI	

measurement of the outcome	4.4 If Y/PY/NI to 4.3: Could assessment of the outcome have been influenced by knowledge of intervention received?	PN	
	4.5 If Y/PY/NI to 4.4: Is it likely that assessment of the outcome was influenced by knowledge of intervention received?	NA	
	**Risk of bias judgement**	**Some concerns**	
**Bias in selection of the reported result**	5.1 Were the data that produced this result analysed in accordance with a pre-specified analysis plan that was finalized before unblinded outcome data were available for analysis?	Y	
	5.2 ... multiple eligible outcome measurements (e.g. scales, definitions, time points) within the outcome domain?	N	
	5.3 ... multiple eligible analyses of the data?	N	
	**Risk of bias judgement**	**Low**	
**Overall bias**	**Risk of bias judgement**	**High**	

**Unique ID**	Choi 2021	**Study ID**	Choi 2021	**Assessor**	Guo
**Ref or Label**	JOURNAL OF WOMEN & AGING	**Aim**	assignment to intervention (the 'intention-to-treat' effect)		
**Experimental**	PT	**Comparator**	N/A	**Source**	Journal article(s)
**Outcome**	GT,CST	**Results**		**Weight**	1

**Domain**	**Signalling question**	**Response**	**Comments**
**Bias arising from the randomization process**	1.1 Was the allocation sequence random?	N	
	1.2 Was the allocation sequence concealed until participants were enrolled and assigned to interventions?	N	
	1.3 Did baseline differences between intervention groups suggest a problem with the randomization process?	NI	
	**Risk of bias judgement**	**High**	
**Bias due to deviations from intended interventions**	2.1.Were participants aware of their assigned intervention during the trial?	NI	
	2.2.Were carers and people delivering the interventions aware of participants' assigned intervention during the trial?	NI	
	2.3. If Y/PY/NI to 2.1 or 2.2: Were there deviations from the intended intervention that arose because of the experimental context?	N	
	2.4 If Y/PY to 2.3: Were these deviations likely to have affected the outcome?	NA	
	2.5. If Y/PY/NI to 2.4: Were these deviations from intended intervention balanced between groups?	NA	
	2.6 Was an appropriate analysis used to estimate the effect of assignment to intervention?	Y	
	2.7 If N/PN/NI to 2.6: Was there potential for a substantial impact (on the result) of the failure to analyse participants in the group to which they were randomized?	NA	
	**Risk of bias judgement**	**Low**	
**Bias due to missing outcome data**	3.1 Were data for this outcome available for all, or nearly all, participants randomized?	NI	
	3.2 If N/PN/NI to 3.1: Is there evidence that result was not biased by missing outcome data?	N	
	3.3 If N/PN to 3.2: Could missingness in the outcome depend on its true value?	N	
	3.4 If Y/PY/NI to 3.3: Is it likely that missingness in the outcome depended on its true value?	NA	
	**Risk of bias judgement**	**Low**	
**Bias in measurement of the outcome**	4.1 Was the method of measuring the outcome inappropriate?	N	
	4.2 Could measurement or ascertainment of the outcome have differed between intervention groups?	N	
	4.3 Were outcome assessors aware of the intervention received by study participants?	N	
	4.4 If Y/PY/NI to 4.3: Could assessment of the outcome have been influenced by knowledge of intervention received?	NA	
	4.5 If Y/PY/NI to 4.4: Is it likely that assessment of the outcome was influenced by knowledge of intervention received?	NA	
	**Risk of bias judgement**	**Low**	
**Bias in selection of the reported result**	5.1 Were the data that produced this result analysed in accordance with a pre-specified analysis plan that was finalized before unblinded outcome data were available for analysis?	Y	
	5.2 ... multiple eligible outcome measurements (e.g. scales, definitions, time points) within the outcome domain?	N	
	5.3 ... multiple eligible analyses of the data?	N	
	**Risk of bias judgement**	**Low**	
**Overall bias**	**Risk of bias judgement**	**Some concerns**	

**Unique ID**	Carrasco-Poyatos 2019	**Study ID**	Carrasco-Poyatos 2019	**Assessor**	Guo
**Ref or Label**	PeerJ 7:e7948	**Aim**	assignment to intervention (the 'intention-to-treat' effect)		
**Experimental**	PT	**Comparator**	No exercise program	**Source**	Journal article(s)
**Outcome**	OLST,TUG	**Results**		**Weight**	1

**Domain**	**Signalling question**	**Response**	**Comments**

**Bias arising from the randomization process**	1.1 Was the allocation sequence random?	Y	A block randomization method was used to allocate participants to the groups with equal sample sizes (PG, MG and CG, n = 20). This randomization method was chosen according to allocation of the specialized senior
	1.2 Was the allocation sequence concealed until participants were enrolled and assigned to interventions?	Y	
	1.3 Did baseline differences between intervention groups suggest a problem with the randomization process?	N	Table 2 defines the characteristics of the participants at baseline for each group.
	**Risk of bias judgement**	**Low**	
**Bias due to deviations from intended interventions**	2.1.Were participants aware of their assigned intervention during the trial?	Y	Owing to the difficulty of blinding the participants and instructors in exercise trials, only the research staff performing the assessment and statistical analysis were blinded to the exercise group assignment
	2.2.Were carers and people delivering the interventions aware of participants' assigned intervention during the trial?	N	
	2.3. If Y/PY/NI to 2.1 or 2.2: Were there deviations from the intended intervention that arose because of the experimental context?	N	
	2.4 If Y/PY to 2.3: Were these deviations likely to have affected the outcome?	NA	
	2.5. If Y/PY/NI to 2.4: Were these deviations from intended intervention balanced between groups?	NA	
	2.6 Was an appropriate analysis used to estimate the effect of assignment to intervention?	Y	
	2.7 If N/PN/NI to 2.6: Was there potential for a substantial impact (on the result) of the failure to analyse participants in the group to which they were randomized?	NA	
	**Risk of bias judgement**	**Low**	
**Bias due to missing outcome data**	3.1 Were data for this outcome available for all, or nearly all, participants randomized?	Y	The proporting of missing follow-up was 11/60
	3.2 If N/PN/NI to 3.1: Is there evidence that result was not biased by missing outcome data?	NA	
	3.3 If N/PN to 3.2: Could missingness in the outcome depend on its true value?	NA	
	3.4 If Y/PY/NI to 3.3: Is it likely that missingness in the outcome depended on its true value?	NA	
	**Risk of bias judgement**	**Low**	
**Bias in measurement of the outcome**	4.1 Was the method of measuring the outcome inappropriate?	N	
	4.2 Could measurement or ascertainment of the outcome have differed between intervention groups?	N	
	4.3 Were outcome assessors aware of the intervention received by study participants?	Y	
	4.4 If Y/PY/NI to 4.3: Could assessment of the outcome have been influenced by knowledge of intervention received?	N	
	4.5 If Y/PY/NI to 4.4: Is it likely that assessment of the outcome was influenced by knowledge of intervention received?	NA	
	**Risk of bias judgement**	**Low**	
**Bias in selection of the reported result**	5.1 Were the data that produced this result analysed in accordance with a pre-specified analysis plan that was finalized before unblinded outcome data were available for analysis?	Y	
	5.2 ... multiple eligible outcome measurements (e.g. scales, definitions, time points) within the outcome domain?	N	
	5.3 ... multiple eligible analyses of the data?	N	
	**Risk of bias judgement**	**Low**	
**Overall bias**	**Risk of bias judgement**	**Low**	

**Unique ID**	Petrofsky 2005	**Study ID**	Carrasco-Poyatos 2019	**Assessor**	Guo
**Ref or Label**	Journal of Applied Research in Clinical and Experimental Therapeutics	**Aim**	assignment to intervention (the 'intention-to-treat' effect)		
**Experimental**	CT with the 6-second abs machine	**Comparator**	N/A	**Source**	Journal article(s)
**Outcome**	FRT	**Results**		**Weight**	1

Domain	Signalling question	Response	Comments
**Bias arising from the randomization process**	1.1 Was the allocation sequence random?	N	
	1.2 Was the allocation sequence concealed until participants were enrolled and assigned to interventions?	N	
	1.3 Did baseline differences between intervention groups suggest a problem with the randomization process?	PN	
	**Risk of bias judgement**	**High**	
**Bias due to deviations from intended interventions**	2.1.Were participants aware of their assigned intervention during the trial?	Y	
	2.2.Were carers and people delivering the interventions aware of participants' assigned intervention during the trial?	PN	
	2.3. If Y/PY/NI to 2.1 or 2.2: Were there deviations from the intended intervention that arose because of the experimental context?	N	
	2.4 If Y/PY to 2.3: Were these deviations likely to have affected the outcome?	NA	
	2.5. If Y/PY/NI to 2.4: Were these deviations from intended intervention balanced between groups?	NA	
	2.6 Was an appropriate analysis used to estimate the effect of assignment to intervention?	PY	
	2.7 If N/PN/NI to 2.6: Was there potential for a substantial impact (on the result) of the failure to analyse participants in the group to which they were randomized?	NA	
	**Risk of bias judgement**	**Low**	
**Bias due to missing outcome data**	3.1 Were data for this outcome available for all, or nearly all, participants randomized?	Y	
	3.2 If N/PN/NI to 3.1: Is there evidence that result was not biased by missing outcome data?	NA	
	3.3 If N/PN to 3.2: Could missingness in the outcome depend on its true value?	NA	

data	3.4 If Y/PY/NI to 3.3: Is it likely that missingness in the outcome depended on its true value?	NA	
	**Risk of bias judgement**	**Low**	
**Bias in measurement of the outcome**	4.1 Was the method of measuring the outcome inappropriate?	PN	
	4.2 Could measurement or ascertainment of the outcome have differed between intervention groups?	Y	
	4.3 Were outcome assessors aware of the intervention received by study participants?	NA	
	4.4 If Y/PY/NI to 4.3: Could assessment of the outcome have been influenced by knowledge of intervention received?	NA	
	4.5 If Y/PY/NI to 4.4: Is it likely that assessment of the outcome was influenced by knowledge of intervention received?	NA	
	**Risk of bias judgement**	**High**	
**Bias in selection of the reported result**	5.1 Were the data that produced this result analysed in accordance with a pre-specified analysis plan that was finalized before unblinded outcome data were available for analysis?	Y	
	5.2 ... multiple eligible outcome measurements (e.g. scales, definitions, time points) within the outcome domain?	N	
	5.3 ... multiple eligible analyses of the data?	N	
	**Risk of bias judgement**	**Low**	
**Overall bias**	**Risk of bias judgement**	**High**	

**Unique ID**	Kang 2012	**Study ID**	Kang 2012	**Assessor**	Guo
**Ref or Label**	Journal of International Academy of Physical Therapy Research	**Aim**	assignment to intervention (the 'intention-to-treat' effect)		
**Experimental**	CT	**Comparator**	No training	**Source**	Journal article(s)
**Outcome**	BBS, Tetrax	**Results**		**Weight**	1

Domain	Signalling question	Response	Comments
**Bias arising from the randomization process**	1.1 Was the allocation sequence random?	Y	hesubjectsthat30personsbetweentheagesof65~80 elderlyparticipatedweredividedintotwogroups randomlyfor8 weeks.
	1.2 Was the allocation sequence concealed until participants were enrolled and assigned to interventions?	PY	
	1.3 Did baseline differences between intervention groups suggest a problem with the randomization process?	N	
	**Risk of bias judgement**	**Low**	
**Bias due to deviations from intended interventions**	2.1.Were participants aware of their assigned intervention during the trial?	Y	
	2.2.Were carers and people delivering the interventions aware of participants' assigned intervention during the trial?	Y	
	2.3. If Y/PY/NI to 2.1 or 2.2: Were there deviations from the intended intervention that arose because of the experimental context?	N	
	2.4 If Y/PY to 2.3: Were these deviations likely to have affected the outcome?	NA	
	2.5. If Y/PY/NI to 2.4: Were these deviations from intended intervention balanced between groups?	NA	
	2.6 Was an appropriate analysis used to estimate the effect of assignment to intervention?	Y	In this study, SPSS(v.17.0) was used to observe the difference between the [illegible]
	2.7 If N/PN/NI to 2.6: Was there potential for a substantial impact (on the result) of the failure to analyse participants in the group to which they were randomized?	NA	
	**Risk of bias judgement**	**Low**	
**Bias due to missing outcome data**	3.1 Were data for this outcome available for all, or nearly all, participants randomized?	Y	
	3.2 If N/PN/NI to 3.1: Is there evidence that result was not biased by missing outcome data?	NA	
	3.3 If N/PN to 3.2: Could missingness in the outcome depend on its true value?	NA	
	3.4 If Y/PY/NI to 3.3: Is it likely that missingness in the outcome depended on its true value?	NA	
	**Risk of bias judgement**	**Low**	
**Bias in measurement of the outcome**	4.1 Was the method of measuring the outcome inappropriate?	N	[illegible] For weight bearing and balance examination, we used [illegible]
	4.2 Could measurement or ascertainment of the outcome have differed between intervention groups?	NI	
	4.3 Were outcome assessors aware of the intervention received by study participants?	N	
	4.4 If Y/PY/NI to 4.3: Could assessment of the outcome have been influenced by knowledge of intervention received?	NA	
	4.5 If Y/PY/NI to 4.4: Is it likely that assessment of the outcome was influenced by knowledge of intervention received?	NA	
	**Risk of bias judgement**	**Some concerns**	
**Bias in selection of the reported result**	5.1 Were the data that produced this result analysed in accordance with a pre-specified analysis plan that was finalized before unblinded outcome data were available for analysis?	Y	
	5.2 ... multiple eligible outcome measurements (e.g. scales, definitions, time points) within the outcome domain?	N	
	5.3 ... multiple eligible analyses of the data?	N	
	**Risk of bias judgement**	**Low**	
**Overall bias**	**Risk of bias judgement**	**Some concerns**	

**Unique ID**	Hosseini 2012	**Study ID**	Hosseini 2012	**Assessor**	Guo
**Ref or Label**	World Applied Sciences Journal	**Aim**	assignment to intervention (the 'intention-to-treat' effect)		
**Experimental**	CT	**Comparator**	No training	**Source**	Journal article(s)
**Outcome**	Y-BT, DGI	**Results**		**Weight**	1

Domain	Signalling question	Response	Comments
**Bias arising from the randomization process**	1.1 Was the allocation sequence random?	Y	Prior to performing the pretest, subjects were randomly divided into three groups.
	1.2 Was the allocation sequence concealed until participants were enrolled and assigned to interventions?	Y	
	1.3 Did baseline differences between intervention groups suggest a problem with the randomization process?	N	
	**Risk of bias judgement**	**Low**	
**Bias due to deviations from intended interventions**	2.1.Were participants aware of their assigned intervention during the trial?	Y	
	2.2.Were carers and people delivering the interventions aware of participants' assigned intervention during the trial?	Y	
	2.3. If Y/PY/NI to 2.1 or 2.2: Were there deviations from the intended intervention that arose because of the experimental context?	N	
	2.4 If Y/PY to 2.3: Were these deviations likely to have affected the outcome?	NA	
	2.5. If Y/PY/NI to 2.4: Were these deviations from intended intervention balanced between groups?	NA	
	2.6 Was an appropriate analysis used to estimate the effect of assignment to intervention?	Y	
	2.7 If N/PN/NI to 2.6: Was there potential for a substantial impact (on the result) of the failure to analyse participants in the group to which they were randomized?	NA	
	**Risk of bias judgement**	**Low**	
**Bias due to missing outcome data**	3.1 Were data for this outcome available for all, or nearly all, participants randomized?	Y	
	3.2 If N/PN/NI to 3.1: Is there evidence that result was not biased by missing outcome data?	NA	
	3.3 If N/PN to 3.2: Could missingness in the outcome depend on its true value?	NA	
	3.4 If Y/PY/NI to 3.3: Is it likely that missingness in the outcome depended on its true value?	NA	
	**Risk of bias judgement**	**Low**	
**Bias in measurement of the outcome**	4.1 Was the method of measuring the outcome inappropriate?	N	
	4.2 Could measurement or ascertainment of the outcome have differed between intervention groups?	N	
	4.3 Were outcome assessors aware of the intervention received by study participants?	Y	
	4.4 If Y/PY/NI to 4.3: Could assessment of the outcome have been influenced by knowledge of intervention received?	N	
	4.5 If Y/PY/NI to 4.4: Is it likely that assessment of the outcome was influenced by knowledge of intervention received?	NA	
	**Risk of bias judgement**	**Low**	
**Bias in selection of the reported result**	5.1 Were the data that produced this result analysed in accordance with a pre-specified analysis plan that was finalized before unblinded outcome data were available for analysis?	Y	
	5.2 ... multiple eligible outcome measurements (e.g. scales, definitions, time points) within the outcome domain?	N	
	5.3 ... multiple eligible analyses of the data?	N	
	**Risk of bias judgement**	**Low**	
**Overall bias**	**Risk of bias judgement**	**Low**	
